# Robust mold fabricated by femtosecond laser pulses for continuous thermal imprinting of superhydrophobic surfaces

**DOI:** 10.1088/2053-1591/ab10c6

**Published:** 2019-04-05

**Authors:** Zhibing Zhan, Erik M Garcell, Chunlei Guo

**Affiliations:** 1 The Institute of Optics, University of Rochester, Rochester, NewYork 14627 United States of America’s Republic of China; 2 Chang chun Institute of Optics, Fine Mechanics, and Physics, Chang chun 130033 People’s Republic of China; 3 These authors contributed equally to this work

**Keywords:** superhydrophobic, thermoplasticimprinting, polymer, pulsed-laserablation

## Abstract

Superhydrophobic surfaces rely on a large number of surface micro/nano structures to increase the roughness of a material. Producing such structures is possible through a multitude of relatively slow methods; however, economic and large scale production of superhydrophobic surfaces require using a fast process on a cheap substrate. Here, we used femtosecond laser processing to fabricate micro and nanostructures on tungsten carbide that we use as a mold to thermally imprint polypropylene sheets. The fabricated tungsten carbide mold was used to imprint more than twenty superhydrophobic polypropylene sheets before mold contamination reduces the surface contact angle below 150°.Using Toluene solution, the mold is subsequently capable of being cleaned of contamination from polypropylene residue and reused for further imprinting. Ninety thermoplastic imprints were conducted using a single tungsten carbide mold with only minimal structural degradation apparent on the micro/nano structured surface.

## 1. Introduction

The hydrophobicity of a material describes the non-wetting characteristics of material surfaces. When a water droplet makes a contact angle greater than 150° with the surface of a material, the material can be considered superhydrophobic [1]. Due to their excellent prospects in the areas of self-cleaning [2–4], anti-icing [5–7], anticorrosion [8–10] and drag reduction [11–13], superhydrophobic surfaces have attracted substantial scientific and commercial attention.

As recently shown by us, femtosecond (fs) laser surface functionalization through micro/nano structure formation is an excellent method to create superhydrophobic structures [14, 15]. Femtosecond laser processing is faster and simpler than many other methods to create superhydrophobic surfaces, such as lithographic patterning [16–18], vertical alignment of nanotubes/nanofibers [19, 20], or sol-gel methods [21, 22]; however, large scale production of superhydrophobic surfaces requires a faster process.

For scaled production, we used thermoplastic imprinting: a replication technique using heated plastics to easily imprint complex mold structures. Unlike other techniques to reproduce superhydrophobic surfaces, such as nanoimprint lithography [23] and nanocasting [24], this process is comprised of only one step. By using a single step replication technique, instead of a direct application technique, or additive/subtractive techniques, we can rapidly increase the production of superhydrophobic surfaces.

The main drawback to the direct imprinting fine structures is that the fine mold structures do not survive multiple prints. A highly wear resistant and extremely hard mold must be utilized to scalably imprint superhydrophobic strcutures. Tungsten carbide is such a material, and is commonly used as tool and die in industry applications. The limiting factor of tungsten carbide is the poor machinability of the material. It’s extreme hardness makes the formation of fine micro and nanostructures virtually impossible by tooling processes [25].We resolve this issue by employing fs laser treatment, which can be used to process most any material [26]. Using fs laser treatment, fine featured tungsten carbide molds were fabricated for our studies.

In this paper, we present a robust imprinting mold developed using femtosecond laser irradiation on tungsten carbide. The fabricated mold is capable of imprinting large numbers of superhydrophobic polypropylene sheets with contact angles above 150° before becoming contaminated. The mold is subsequently capable of being cleaned of contamination from polypropylene with minimal structural degradation. The ability to retain micro/nanostructures after batch imprinting and cleaning allows for more continuous use of the mold, and subsequently less material and fabrication cost than previous superhydrophobic imprinting efforts.

## 2. Methods

Used in our studies as the imprinting mold, a 25.4 mm squared, 2 mm thick, tungsten carbide (WC) sample is raster scanned using 65 fs linearly polarized pulses from a Ti-sapphire laser system at a single pulse fluence of 9.8 J cm^−2^. The Ti-sapphire fs laser system was operated at a 1 kHz repetition rate at a central wavelength of 800 nm. Raster scanning was performed at a speed of 0.5 mm s^−1^ with an interline periodicity of 100 um, equivalent to the size of the focused laser beam’s diameter on the WC surface. The WC used is comprised of 6% cobalt. Tungsten carbide was selected as the mold material due to its hardness and industry relevance. 35 mm squared, 1.6 mm thick, sheets of polypropylene (PP) were used as the imprint material due to PP’s low cost and commercial relevance.

Imprinting was performed on a hydraulic press with heated platens. Imprinting was performed by first contacting the WC mold and PP sheets between the heated platens of the hydraulic press and subsequently heating the platens to a temperature of 115 °C. After two minutes, the pressure of the hydraulic press is increased until an applied force of 13.37 kN is achieved (20.57 MPa imprint pressure). The force applied by the hydraulic press was calibrated using a button load cell. After holding at the set temperature and pressure for three minutes, the pressure is released and the PP is removed from the WC stamp. No solution or additive is used on the WC to assist in demolding. Before imprinting, the prepared WC sample was ultrasonically cleaned in a solution of distilled water for 10 s to remove any loose surface nanoparticles.

The surface structures of both the laser irradiated WC and the imprinted PP were studied using a scanning electron microscope (SEM) and a UV laser-scanning confocal microscope (UV-LSCM). Contact angle of water droplets on the imprinted PP were measured by the sessile drop method on a Kino brand static optical contact angle meter.

There was no noticeable difference in contact angle or sliding angle for parallel or perpendicular directionalities, with respect to the laser ablated grooves.

## 3. Results and discussion

**Figure 1:**
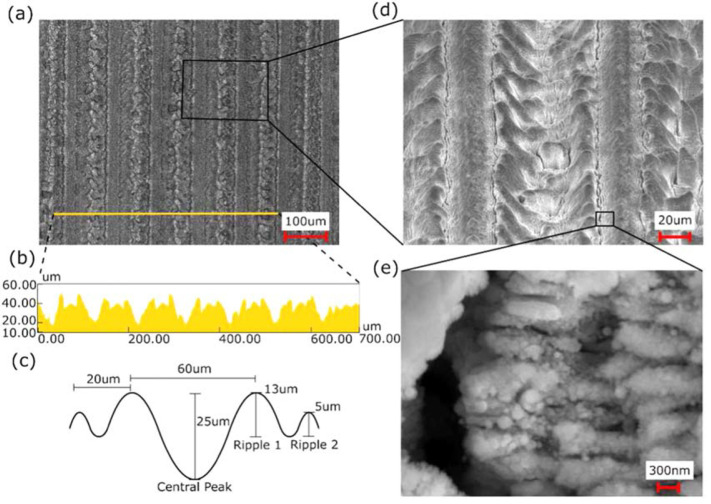
SEM and height profile of the laser irradiated tungsten carbide (WC) mold before imprinting. (a) Image of the raster scanned lines on the WC surface. (b) Surface profile for the line shown in image (a). (c) Graphic representation and average dimensions of the profile of a single raster scanned line. (d) Zoomed in image of the raster scanned lines. (e) Further zoomed in image showing LIPSSs structures formed on the WC surface.

After raster scanning of the WC surface using a pulsed fs laser, a series of 100 umspaced ablation lines are formed on its surface (figures 1(a) and (b)). At the center of the raster scanned line, where the Gaussian shaped laser beam’s intensity is largest, a 25 umdeep depression is formed. Oneither side of this depression is a series of two ripples that decrease in amplitude moving away from the intensity maxima. The amplitude of the larger ripple is on average 13 um, while the smaller ripple is on average 5 um (figure 1(c)). These side ripples, along with the lack of uniformity of the sidewalls of the central depression, are indicative of melting and resolidification dynamics (figure 1(d)) [27]. The absorbed laser energy for material adjacent to the laser beam’s intensity maxima, not having superseded the material’s ablation threshold, diffused into the material’s lattice causing melting and subsequent formation and solidification of capillary waves [15]. While melt dynamics are clearly evident from the rippling form of these structures, ablation dynamics are also clearly evident from the layer of redeposited nanoparticle dust deposited on the material’s surface, as observed before ultrasonic cleaning. Interestingly, forming on and along the center depression and adjacent ripples are parallel periodic lines known as laserinduced periodic surface structures (LIPSSs) (figure 1(e)). The LIPSSs on all the aforementioned structures are formed with an average periodicity of 580 nm. These structures are commonly thought to be formed by the interference between incident laser light and surface scattered electromagnetic waves [28]. These structures have been shown to form both above and below the ablation threshold of a material [29], which would explain their formation in both the ablation and melted areas of the raster scanned surface.

**Figure 2:**
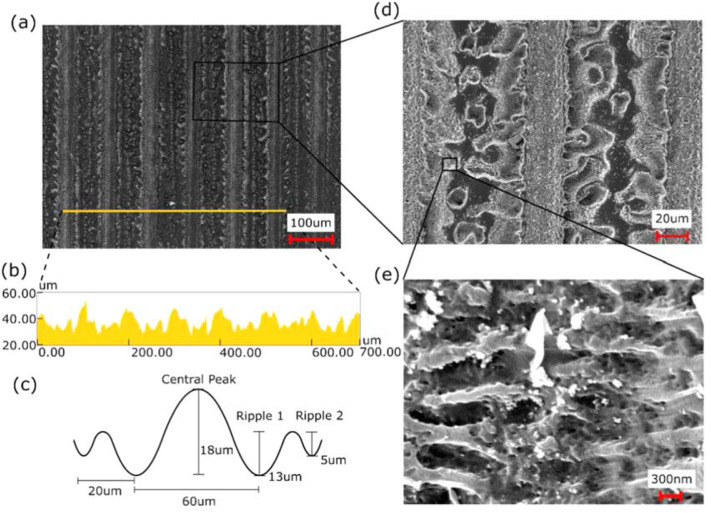
SEM and height profile of a typical imprinted polypropylene (PP) surface. (a) Image of the imprinted raster scanned lines from the tungsten carbide mold surface on the 10th imprinted PP sheet. (b) Surface profile for the line shown in image (a). (c) Graphic representation and average dimensions of the profile of a single imprinted raster scanned line. (d) Zoomed in image of the imprinted raster scanned lines. (e) Further zoomed in image showing LIPSSs structures imprinted on the PP surface.

Using an applied pressure of 20.57 MPa and a temperature of 115 °C, we use the laser treated WC to imprint PP sheets. Imprinting with these parameters yielded a maximal hydrophobic response of 162°. Further increasing pressures or temperatures caused deformation of the PP substrait and significant surface contamination after demolding, with no increase to the material’s hydrophobic response. Figure 2 is a representative sample of the imprinted PP surface. Using these imprint parameters, a clear negative of the WC structures are formed (figure 2(b)). The height of the imprinted center depression is on average 18 um, a 28% decrease from the 25 umdeep structures formed on the WC mold. The imprinted first and second peripheral ripples are equivalent to the analogous WC mold structures, being on average 13 umand 5 umin amplitude respectively (figure 2(c)). LIPSS are also clearly imprinted on the PP surface, having the same 580 nmperiod as the WC mold (figure 2(e)).

**Figure 3:**
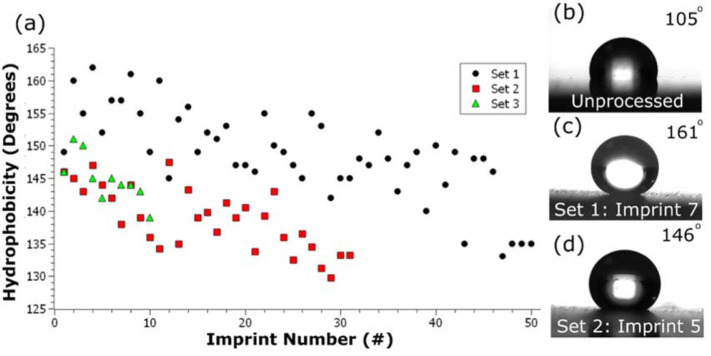
Plot and photos of hydrophobicity of polypropylene (PP) surfaces. (a) Contact angle versus imprint number for imprinted PP surfaces. (b)–(d) Photos of water droplets on an unprocessed PP sample, an imprinted PP surface from set 1 and an imprinted PP surface from set 2, respectively.

To test the robustness of the processed WC mold, 50 imprints were performed first, which we will call set 1. After set 1, two rounds of cleaning and further imprinting were performed, which we will call sets 2 and 3, respectively. Tracking the hydrophobicity of all imprinted PP samples (figure 3), it can be seen that the highest initial achievable hydrophobicity is 162°, and that hydrophobicity trends down with increased imprint repetition. The hydrophobicity of set 1 trends as: *θ* = −0.35x + 158. Where *θ* and *x* stand for the water droplet contact angle and imprint number, respectively. For set 1, imprinted PP trends superhydrophobic up to 22 imprints, after which hydrophobicity continues to decrease. The decrease in hydrophobicity correlates to the prevalence and increase of PP contamination on the surface of the WC mold. This contamination is thought to reduce the imprinted surface contact angle by reducing the roughness of the imprinted surface.

Polypropylene contamination on the mold inhibits the replication of the micro and nano structures beneath these contaminated areas. The reduction in micro/nanostrcuture imprinting results in a surface with a lower overall surface roughness. While the PP residue does add topography to the mold surface, this new PP topography is less hierarchical than the laser ablated mold surface and so reduces the overall surface roughness. This observation is best understood when considering the Wenzel model for hydrophobicity. A simple model for hydrophobicity, the Wenzel model states that a decrease in the roughness ratio (the ratio of the true area to the apparent area) of a surface will result in a proportional decrease to that surface’s hydrophobicity [30]. Thus, the reduction in the surface roughness of the imprinted surface will cause a reduction in the imprinted plastic’s hydrophobic response.

**Figure 4:**
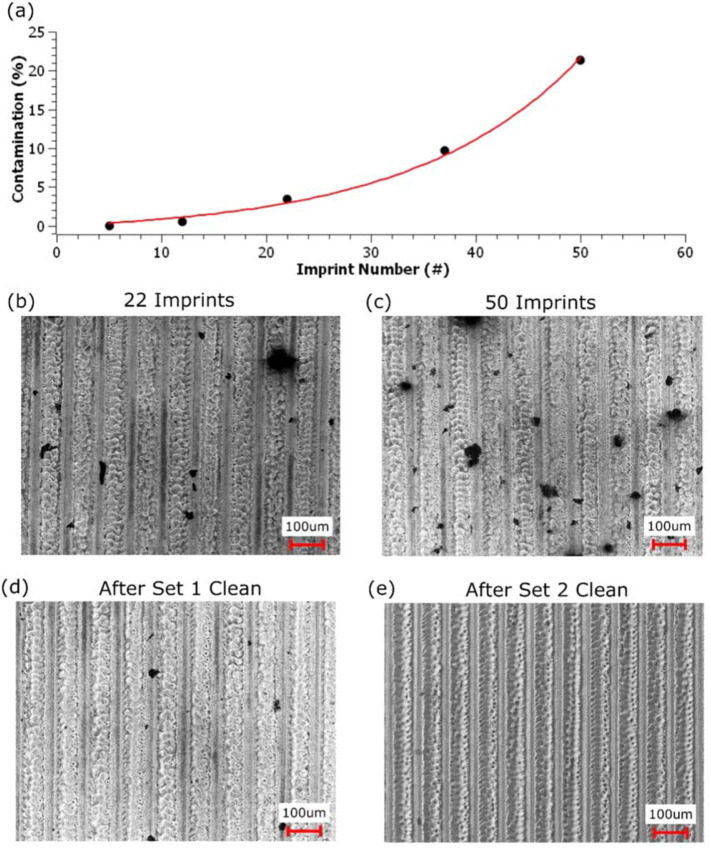
Plot and photos of polypropylene (PP) contamination on the tungsten carbide (WC) mold surface. (a) Plot of percentage area contamination on the WC surface versus imprint number. (b)–(e) show images of the WC sample after 22 imprints, 55 imprints, cleaning after set 1, and cleaning after set 2, respectively.

Contamination of the surface occurs from PP adhesion during the demolding process. As the number of imprints increase, the size and frequency of contamination on the WC surface increases (figures 4(a)–(c)). The percentage of total surface contamination is found to increase at a rate of about *e*^(0.062*^x^)^, where *x* is the imprint number (figure 4(a)). After 50 imprints, the stamp was cleaned using Toluene, a common solvent for PP. After cleaning the sample for 30 min at 80 °C, all particles with diameters below 20 umsize are removed, leaving only large contaminates behind (figure 4(d)). Following this cleaning, the WC stamp was used to perform an additional 30 imprints. The hydrophobic response of the imprinted PP surfaces of set 2 trend similarly to that of set 1 but are consistently 10° lower, on average. After set 2, the mold was again cleaned in Toluene, but this time for one hour at 120 °C, and subsequently used to imprint an additional 10 stamps. The mold after this second cleaning is almost entirely free of PP residue (figure 4(e)). This third round of imprinting resulted in a slightly elevated maximal hydrophobic response, 151° for set 3 versus 146° for set 2, but again trends 10° lower than set 1, on average. After set 3, one additional imprint was conducted to test the sliding angle of the imprinted surface. It is found that after 90 imprints, the sliding angle of the imprinted PP surface is 4 degrees. This low sliding angle further demonstrates that the surface of the imprinted PP is best characterized by the Wenzel model for hydrophobicity.

**Figure 5:**
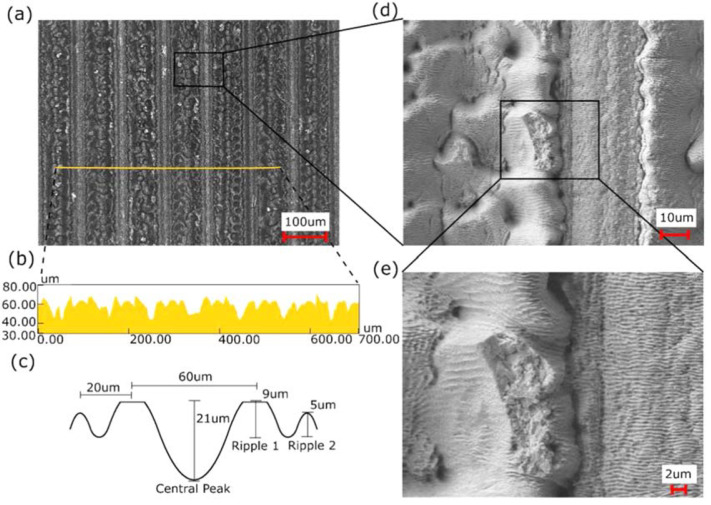
SEM and height profile of the laser irradiated tungsten carbide (WC) mold after 90 imprints. (a) Image of the raster scanned lines on the WC surface. (b) Surface profile for the line shown in image (a). (c) Graphic representation and average dimensions of the profile of a single raster scanned line. (d) Zoomed in image of the raster scanned lines. (e) Further zoomed in image showing LIPSSs structures formed on the WC surface.

The reduction in the initial achievable hydrophobicity between set 1 and sets 2 and 3 is likely due to damage of the mold structures. First observed after completing set 2, damage can be found on the tips of the structures produced on the WC mold (figure 5). Recalling the structures formed on our WC mold surface from figure 1, after 90 imprints it is evident that the tips of what we label ‘ripple 1’ have been damaged. After 90 imprints, the size of ripple 1 has been reduced from an initial height of 13 umto 9 umon average, making it more level with adjacent structures (figure 5(c)). Ripple 1, being the more prominent of the two ripple structures on the WC surface, experiences increased pressure, causing the tip of ripple 1 structures to be ground down. This change in height of ripple 1 decreases the overall surface roughness of the WC mold, and subsequently the imprinted PP surface. According to the Wenzel model for hydrophobicity, the reduction in the surface roughness of the mold between sets 1 and 2 and 3, caused by the wearing down of the ripple 1 structures, is the cause of the reduction in the imprinted plastic’s hydrophobic response. Between sets 2 and 3, no further change is observed in the structure’s morphology.

Besides this work, several other works have been conducted to replicate superhydrophobic structures. Two examples are, Bekesi *et al* 2010 [31] using injection molding and George *et al* 2018 [32] employing soft lithography, resulted in a contact angle above 160° for PP and 156° for Polydimethylsiloxane (PDMS), respectively. These works yield contact angles comparable to what have been demonstrated in this manuscript, but do not demonstrate the repeatability or durability of their processes. Few works have demonstrated the robustness of their processes as has been demonstrated in this manuscript. One notable exception is Yan *et al* 2017 [33] which employed a steel mold to imprint microcraters onto silicone rubber. Starting with a contact angle of 151.5°, after 50 imprints the mold was able to maintain a contact angle of 150.2°. While our molds can initially imprint PP with a contact angle of up to 162°, after 50 imprints this contact angle is reduced to nearly 135°, much lower than what is demonstrated in the work Yan *et al* 2017 [33]. However, unlike other works we have been able to demonstrate that the mold structures we employ are capable of being cleaned and reused, which significantly restores the hydrophobicity of the PP surface up to 151°.

After an initial drop in hydrophobic response of 10°, caused by a wearing down of the outlying mold’s surface features, the WC mold is capable of being used and cleaned repeatably without further drop in hydrophobic response. Such a mold, capable of being cleaned without the need for replacement would drastically increase the scalability and lower the cost of production for industry scale production of superhydrophobic structured polymer surfaces.

## 4. Conclusion

A robust mold for the imprinting of superhydrophobic structures on polymer surfaces has been presented. The laser-fabricated WC mold here demonstrated is capable of imprinting up to 22 PP sheets with water droplet contact angles above 150°. Once surface contamination of the mold causes imprints to fall below superhydrophobic levels, the WC mold is capable of being cleaned and used for hydrophobic replication repeatably. An observed drop of 10° hydrophobicity between the pristine mold and mold after 50 imprints and a first cleaning can be attributed to slight structural degradation of the surface that, out to 90 imprints and an additional cleaning, does not continue to degrade.

## References

[1] Wang S and Jiang L 2007 Adv. Mater. 19 3423–4

[2] Fürstner R, Barthlott W, Neinhuis C and Walzel P 2005 Langmuir 21 956–61 15667174 10.1021/la0401011

[3] Bhushan B, Jung YC and Koch K 2009 Philos. Trans. RoyalSoc. A 367 1631–72 10.1098/rsta.2009.0014 19376764

[4] Bhushan B, Jung YC and Koch K 2009 Langmuir 25 25 3240–8 19239196 10.1021/la803860d

[5] Cao L, Jones AK, Sikka VK, Wu J and Gao D 2009 Langmuir 25 12444–8 19799464 10.1021/la902882b

[6] Farhadi S, Farzaneh M and Kulinich S 2011 Appl. Surf. Sci. 257 6264–9

[7] Antonini C, Innocenti M, Horn T, Marengo M and Amirfazli A 2011 Cold Reg. Sci. Technol. 67 58–67

[8] Isimjan TT, Wang T and Rohani S 2012 Chem. Eng. J. 210 182–187

[9] Chen Y, Chen S, Yu F, Sun W, Zhu H and Yin Y 2009 Surf. Interface Anal. 41 872–7

[10] Zhang H, Yang J, Chen B, Liu C, Zhang M and Li C 2015 Appl. Surf. Sci. 359 905–10

[11] Bhushan B and Jung YC 2011 Prog. Mater Sci. 56 1–108

[12] Daniello RJ, Waterhouse NE, Rothstein JP (2009). Phys. Fluids.

[13] Truesdell R, Mammoli A, Vorobieff P, vanSwol F and Brinker CJ 2006 Phys. Rev. Lett. 97 044504 16907578 10.1103/PhysRevLett.97.044504

[14] Vorobyev AY and Guo C 2015 J. Appl. Phys. 117 033103

[15] Vorobyev AY and Guo C 2012 Laser Photonics Rev. 7 385–407

[16] Shiu JY, Kuo CW, Chen P and Mou CY 2004 Chem. Mater. 16 561–4

[17] Pozzato A, Zilio SD, Fois G, Vendramin D, Mistura G, Belotti M, Chen Y and Natali M 2006 Microelectron.Eng. 83 884–8 Micro-and Nano-Engineering MNE 2005

[18] Liu B, He Y, Fan Y and Wang X 2006 Macromol. Rapid Commun. 27 1859–64

[19] Lau KKS, Bico J, Teo KBK, Chhowalla M, Amaratunga GAJ, Milne WI, McKinley GH and Gleason KK 2003 Nano Lett. 3 1701–5

[20] Jin M, Feng X, Feng L, Sun T, Zhai J, Li T and Jiang L 2005 Adv. Mater 17 1977–81

[21] Rao AV, Latthe SS, Mahadik SA and Kappenstein C 2011 Appl. Surf. Sci. 257 5772–6

[22] Shirtcliffe NJ, McHale G, Newton MI and Perry CC 2003 L angmuir 19 5626–31

[23] Radha B, Lim SH, Saifullah MS and Kulkarni GU 2013 Sci. Rep. 3 31078 10.1038/srep01078 PMC3584315 23446801

[24] Sun M, Luo C, Xu L, Ji H, Ouyang Q, Yu D and Chen Y 2005 L angmuir 21 8978–81 10.1021/la050316q 16142987

[25] Ottersbach M and Zhao W 2016 Proc. CIRP 46 416–9

[26] Joglekar A, Liu H, Spooner G, Meyhöfer E, Mourou G and Hunt A 2003 Appl. Phys. B 77 25–30

[27] Bonse J, Bachelier G, Siegel J, Solis J (2006). Phys. Rev. B.

[28] Bonse J, Höhm S, Kirner SV, Rosenfeld A and Krüger J 2017 IEEE J. Sel. Top. Quantum Electron. 23 9000615

[29] Reyes-Contreras A, Camacho-López M, Camacho-López S, Olea-Mejía O, Esparza-García A, Nuelos Muñetón JGB and Camacho-López MA 2017 Opt. Mater. Express 7 1777–86

[30] Lafuma A and Quéré D 2003 Nat. Mater. 2 457 12819775 10.1038/nmat924

[31] Bekesi J, Kaakkunen J, Michaeli W, Klaiber F, Schoengart M, Ihlemann J and Simon P 2010 Appl. Phys.A 99 691–5

[32] George JE, Unnikrishnan V, Mathur D, Chidangil Sand George SD 2018 Sens. Actuator B-Chem. 272 485–93

[33] Yan Z, Liang X, Shen Hand Liu Y 2017 IEEE Trans.Dielectr.Electr.Insul. 24 1743–50

